# Identification of risk factors for hospital admission using multiple-failure survival models: a toolkit for researchers

**DOI:** 10.1186/s12874-016-0147-x

**Published:** 2016-04-26

**Authors:** Leo D. Westbury, Holly E. Syddall, Shirley J. Simmonds, Cyrus Cooper, Avan Aihie Sayer

**Affiliations:** MRC Lifecourse Epidemiology Unit, University of Southampton, Southampton, UK; NIHR Southampton Biomedical Research Centre, University of Southampton and University Hospital Southampton NHS Foundation Trust, Southampton, UK; NIHR Musculoskeletal Biomedical Research Unit, University of Oxford, Oxford, UK; Academic Geriatric Medicine, Faculty of Medicine, University of Southampton, Southampton, UK; NIHR Collaboration for Leadership in Applied Health Research and Care, Wessex, Southampton, UK; Institute for Ageing and Institute of Health & Society, Newcastle University, Newcastle, UK

**Keywords:** Medical statistics, Epidemiological methods, Cohort studies, Hospital admissions, Multiple-failure, Survival analysis, Risk factor, Older people

## Abstract

**Background:**

The UK population is ageing; improved understanding of risk factors for hospital admission is required. Linkage of the Hertfordshire Cohort Study (HCS) with Hospital Episode Statistics (HES) data has created a multiple-failure survival dataset detailing the characteristics of 2,997 individuals at baseline (1998–2004, average age 66 years) and their hospital admissions (regarded as ‘failure events’) over a 10 year follow-up. Analysis of risk factors using logistic regression or time to first event Cox modelling wastes information as an individual’s admissions after their first are disregarded. Sophisticated analysis techniques are established to examine risk factors for admission in such datasets but are not commonly implemented.

**Methods:**

We review analysis techniques for multiple-failure survival datasets (logistic regression; time to first event Cox modelling; and the Andersen and Gill [AG] and Prentice, Williams and Peterson Total Time [PWP-TT] multiple-failure models), outline their implementation in Stata, and compare their results in an analysis of housing tenure (a marker of socioeconomic position) as a risk factor for different types of hospital admission (any; emergency; elective; >7 days). The AG and PWP-TT models include full admissions histories in the analysis of risk factors for admission and account for within-subject correlation of failure times. The PWP-TT model is also stratified on the number of previous failure events, allowing an individual’s baseline risk of admission to increase with their number of previous admissions.

**Results:**

All models yielded broadly similar results: not owner-occupying one’s home was associated with increased risk of hospital admission. Estimated effect sizes were smaller from the PWP-TT model in comparison with other models owing to it having accounted for an increase in risk of admission with number of previous admissions. For example, hazard ratios [HR] from time to first event Cox models were 1.67(95 % CI: 1.36,2.04) and 1.63(95 % CI:1.36,1.95) for not owner-occupying one’s home in relation to risk of emergency admission or death among women and men respectively; corresponding HRs from the PWP-TT model were 1.34(95 % CI:1.15,1.56) for women and 1.23(95 % CI:1.07,1.41) for men.

**Conclusion:**

The PWP-TT model may be implemented using routine statistical software and is recommended for the analysis of multiple-failure survival datasets which detail repeated hospital admissions among older people.

## Background

The UK population is ageing [1]; improved understanding of lifecourse risk factors for hospital admission is required to identify subgroups of the population who are at increased risk of hospital admission, and to inform the development of intervention strategies to delay or prevent admissions to hospital [2].

Recent linkage between the Hertfordshire Cohort Study (HCS) database and routinely collected Hospital Episode Statistics (HES) data has yielded a complex dataset which comprises baseline information on socio-demographic, lifestyle and clinical characteristics of 2,997 community-dwelling men and women (average age 66 years at baseline 1998–2004) together with details of all inpatient hospital admissions over a 10 year follow-up period [3]. HCS is the first UK birth cohort study to link with HES data but other well established UK cohorts [4, 5] have the potential to do so. Cohort study databases that have been linked with HES data are a rich resource for the investigation of risk factors for hospital admission among older men and women but require sophisticated statistical analysis techniques if they are to be fully explored.

A dataset which contains information about hospital admission histories for study participants may be referred to as a ‘multiple-failure survival dataset’. In this context, a hospital admission is regarded as a ‘failure event’; study participants may experience none, one, or many failure events during the study follow-up period. Statistical analysis techniques for multiple-failure survival datasets are well established [6, 7] but little applied in medical research owing to their complexity. We are not aware of any previous publications that have used multiple-failure survival analysis techniques to analyse risk factors for hospital admission among community-dwelling older people in the UK.

The objectives of this paper are to provide researchers with a ‘toolkit’ for the analysis of multiple-failure survival datasets by: reviewing suitable statistical analysis techniques; outlining their implementation using the Stata statistical software package; and contrasting the application of these techniques to an analysis of the association between housing tenure, a marker of socioeconomic position, and different types of hospital admission in the linked HCS-HES dataset.

## Structure of the linked HCS-HES multiple-failure survival dataset

### Hertfordshire cohort study

The Hertfordshire Cohort Study has been described in detail previously [8]. In brief, the cohort comprises 1579 men and 1418 women born in Hertfordshire between 1931 and 1939 and who still lived in the county between 1998 and 2004 when they participated in a nurse administered home interview and attended a clinic for detailed physiological investigations. The HCS database includes detailed information on study participants’ socio-demographic, lifestyle and clinical characteristics. The study had ethical approval from the Hertfordshire and Bedfordshire Local Research Ethics Committee. All participants gave signed consent for the investigations they underwent in clinic and for researchers to access their medical records in the future. Investigations on participants were conducted in accordance with the principles expressed in the Declaration of Helsinki. Participants are flagged for continuous notification of death on the Central Register at the NHS Information Centre.

### Linkage with HES data

Permission to obtain a HES extract for HCS participants covering the period 01/04/98–31/03/10 was granted by the Ethics and Confidentiality Committee of the National Information Governance Board. Linkage of the HCS database with Hospital Episode Statistics (HES) data has been described in detail previously [3]; the HES extract included information on date and method of admission (elective or emergency), primary diagnoses coded to ICD-10, and date of discharge. A total of 8687 admissions was identified among 2161 HCS participants after their date of HCS baseline clinic but before 31/3/10; 836 had no admissions. In total, 275 members of the cohort had died during the follow-up period, 21 without admission, 127 during an admission and 127 after being discharged alive.

### Extract of the HCS-HES multiple-failure survival dataset

In this paper, we regard hospital admission or death as failure events. Accordingly, by the end of follow-up, a participant will have experienced one of the following:

- no admissions and survived (no failures);
- no admissions and died (one failure);
- one admission and survived (one failure);
- two or more admissions and survived (multiple failures);
- one or more admission and died (multiple failures).

**Fig. 1:**
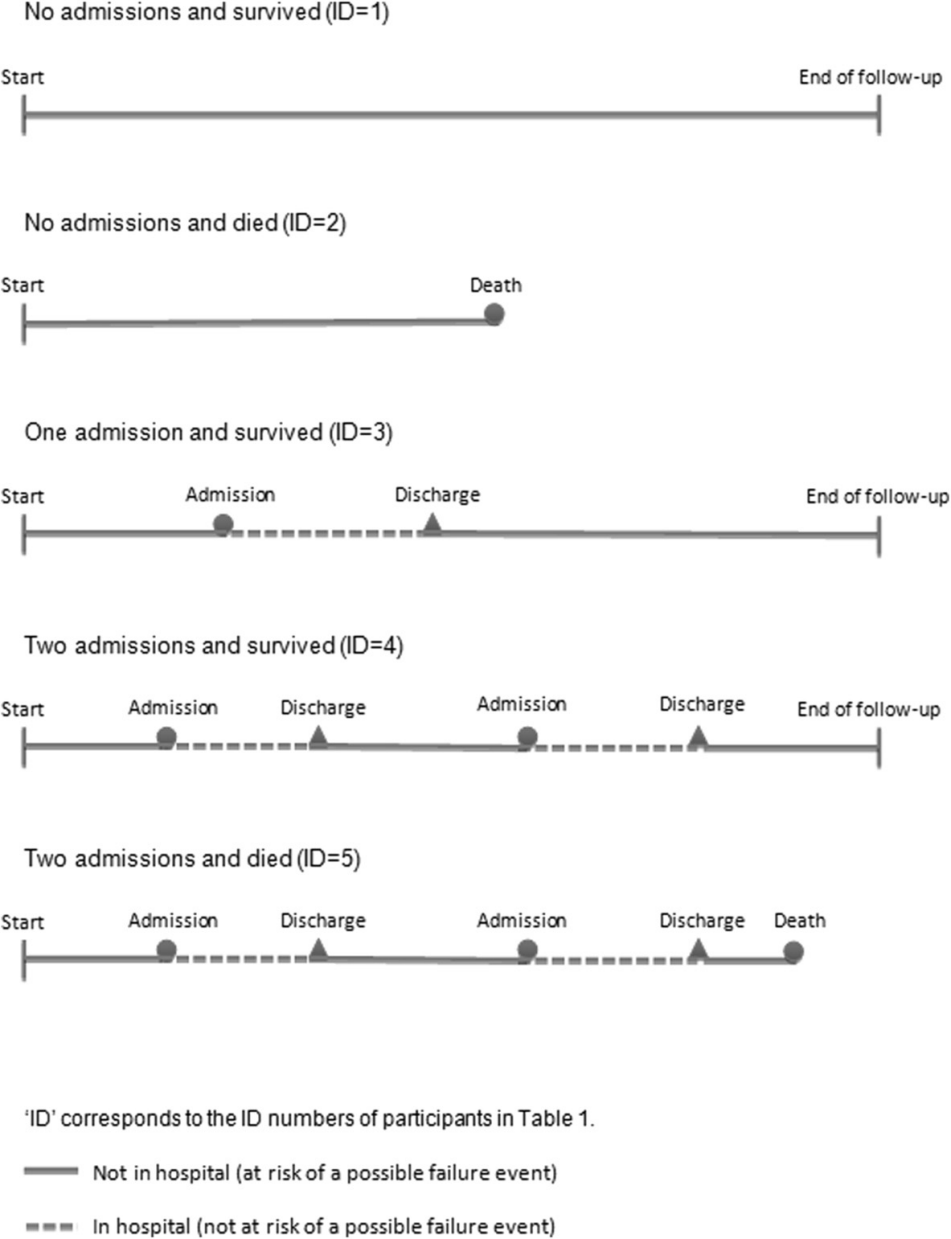
Illustration of potential patterns of follow-up

These five potential patterns of follow-up are illustrated in Fig. 1; extracts from the HCS-HES database which correspond to participants with these patterns of follow-up are listed in Tables 1 and 2 . Extract 1 lists admission and discharge dates for all hospital admissions experienced by the five indexed participants along with dates of death. In order to implement survival analysis techniques, the HCS-HES data were mapped from this initial format to a multiple-failure survival dataset which comprised separate periods during which study participants were at risk of hospital admission or death (Extract 2). Participants were not regarded as being at risk of a subsequent failure event whilst they were in hospital; the failure date for individuals who died whilst in hospital was coded as the date they were admitted to hospital.

**Table 1:** Extracts from the HCS-HES database corresponding to participants with different patterns of follow-up

Extract 1: Dates of clinic visit, admission, discharge and death in the linked HCS-HES admissions file					
ID	Gender	Clinic	Admission date	Discharge date	Date of death
1	Male	17-Sep-00	.	.	.
2	Female	14-Jan-00	.	.	23-Jun-07
3	Female	23-Feb-01	06-Mar-06	09-Mar-06	.
4	Male	20-May-01	08-Apr-03	27-Apr-03	.
4	Male	20-May-01	24-May-03	05-Jun-03	.
5	Female	18-Sep-00	28-Jan-04	15-Feb-04	01-Mar-05
5	Female	18-Sep-00	13-Jan-05	16-Jan-05	01-Mar-05

Information in the column 'Gender' is obtained from the HCS dataset. More information in the HCS dataset such as participants’ socio-demographic, lifestyle and clinical characteristics at baseline is also included in the linked HCS-HES admissions file

**Table 2:** Extracts from the HCS-HES database corresponding to participants with different patterns of follow-up

Extract 2: Intervals at risk and indicators of failure events in the linked HCS-HES survival file								
ID	Gender	Clinic	Start	End	Admission	Death	Failure	Risk_set
1	Male	17-Sep-00	17-Sep-00	31-Mar-10	0	0	.	1
2	Female	14-Jan-00	14-Jan-00	23-Jun-07	0	1	1	1
3	Female	23-Feb-01	23-Feb-01	06-Mar-06	1	0	1	1
3	Female	23-Feb-01	09-Mar-06	31-Mar-10	0	0	.	2
4	Male	20-May-01	20-May-01	08-Apr-03	1	0	1	1
4	Male	20-May-01	27-Apr-03	24-May-03	1	0	1	2
4	Male	20-May-01	05-Jun-03	31-Mar-10	0	0	0	3
5	Female	18-Sep-00	18-Sep-00	28-Jan-04	1	0	1	1
5	Female	18-Sep-00	15-Feb-04	13-Jan-05	1	0	1	2
5	Female	18-Sep-00	16-Jan-05	01-Mar-05	0	1	1	3

'ID' represents the individual the data correspond to

‘Clinic’ specifies the date of the HCS baseline clinic. This was the date that each individual first entered the study and, therefore, the first time they were regarded as being at risk of admission/death

'Start' specifies the beginning of the time interval when the individual was at risk of admission/death. This can be the date the individual attended the clinic or the date of discharge from a previous admission

'End' specifies the end of the time interval when the individual was at risk of admission/death. This can be the end of follow-up or the date of an admission/death

'Admission' and 'Death' indicate the occurrence of these events at the end of the risk interval and 'Failure' indicates the occurrence of admission/death

In Table 1 and 2, ID numbers and dates of admission, discharge and death are fictitious and are purely for illustrative purposes

### Overview of statistical analysis techniques for multiple-failure survival datasets

This section outlines a range of progressively more sophisticated analysis techniques for multiple-failure survival datasets: logistic regression; the time to first event Cox proportional hazards model; the Andersen and Gill (AG) model; and the Prentice, Williams and Peterson (PWP-TT) Total Time model.

### Logistic regression

Logistic regression is a simple technique that can be used to analyse the association between the odds of *ever *having a failure event in relation to a range of risk factors; this approach has been used to investigate whether hospital admission is associated with individual characteristics such as age, gender and socioeconomic factors [9, 10]. Predictive models such as the Patients at Risk of Re-Hospitalisation (PARR) [11] and the Scottish Patients at Risk of Readmission and Admission (SPARRA) [12] have been developed using logistic regression.

A logistic regression approach could be applied to the analysis of multiple-failure survival datasets by reducing each individual’s hospital admission and mortality history to a binary variable which simply indicates whether or not an admission or death was ever experienced. However, this approach is simplistic as it takes no consideration of the different times from baseline to an individual’s first admission and, moreover, ignores all admissions after the first.

### Time to first event Cox proportional hazards model

The Cox proportional hazards model can be used to analyse the association between time to a failure event and a range of risk factors; a comprehensive introduction to this survival analysis technique is provided by Hosmer and Lemeshow [13]. When applied to a multiple-failure survival dataset, this approach only examines the association between the characteristics of study participants and the time to their first failure event [14]. This approach was used to investigate risk factors for emergency hospital admission among 2,849,381 patients from the QResearch cohort and the Clinical Practice Research DataLink cohort [15].

A Cox proportional hazards model is more sophisticated than logistic regression because it considers the *time* to the first failure event. However, it still wastes information because failures after the first are disregarded; this can result in erroneous conclusions about the association between risk factors and failure events. For example, one study showed that sunscreen treatment was not associated with basal cell carcinoma (BCC) in a time to first event analysis but was associated with a lower risk of BCC when recurrences of the illness were also incorporated in the analysis as failure events [16].

A standard Cox proportional hazards model cannot be used to analyse repeated events in multiple-failure survival datasets because failure times from the same individual are likely to be correlated and, therefore, the assumption of independent observations required by the Cox model would not be satisfied [14].

### Multiple-failure survival models

Complex multiple-failure survival analysis techniques are available; we outline the Andersen and Gill (AG) and the Prentice, Williams and Peterson Total Time (PWP-TT) models. The advantage of these techniques compared to both logistic regression and time to first event Cox modelling is that individuals’ failure events after the first are incorporated. In addition, the AG and PWP-TT models are variance correction models which account for within-subject correlation of failure times unlike a standard Cox model [17].

There are similarities between the AG and the PWP-TT models. Firstly, both approaches estimate hazard ratios for the association between risk factors and failure events. Secondly, in common with standard Cox modelling, these techniques assume proportional hazards [18] meaning that the difference in the risk of failure in relation to a risk factor is time independent; this assumption can be assessed by investigating the relationship between the scaled Schoenfeld residuals and functions of time, either by graphical examination or significance testing [16].

However, there is an important difference between these techniques which is particularly relevant when applied to the analysis of a multiple-failure dataset for hospital admissions. In the AG model, the underlying risk of failure is regarded as the same for each event within an individual whereas the PWP-TT model allows this underlying risk to vary [14]. In the context of hospital admission among older people, it is reasonable to expect that risk of admission will increase with the accumulated number of previous admissions; stratified analyses illustrated that this was the case in the HCS-HES dataset (data not shown). Therefore, we recommend that the PWP-TT model is better suited than the AG model for the analysis of risk factors for hospital admission among older people as it allows the underlying risk of admission to increase with the number of accrued admissions. The PWP-TT model is in essence a variance corrected stratified Cox model, with stratification on the number of previous failure events. An introduction to the mathematical basis of the AG and PWP-TT models is beyond the scope of this paper but has been published previously [19, 20].

### Implementation in Stata

Table 3 provides the Stata command syntax for implementation of the analysis techniques described above; variable names included in the command syntax are as shown in Extract 2. An online resource [21] provided guidance on how to implement the multiple-failure time models using Stata in a general setting. We did not implement logistic regression analysis because this technique is routinely available in statistical software packages and, more importantly, we recommend that it is an over-simplistic analysis for a multiple-failure survival dataset such as the HCS-HES dataset.

**Table 3:** Stata specification for each model

Model type	Commands to convert dataset as formatted in Extract 2 to a format suitable for model fitting	Description of commands to convert dataset	Commands to fit models	Description of commands to fit models
Time to first event Cox	*stset End, failure(Failure==1) id(ID) origin(time Clinic) enter(time Clinic) scale(365.25) time0(Start)*	*origin(time Clinic) *and *enter(time Clinic) *specify that the individual first becomes at risk after the clinic date	*stcox varlist*	*varlist* represents a list of predictor variables in the model
Andersen and Gill	*stset End, failure(Failure==1) id(ID) origin(time Clinic) enter(time Clinic) scale(365.25) time0(Start) exit(time.)*	*scale(365.25) *ensures that time is measured in years as opposed to days	*stcox varlist, efron robust*	*efron *specifies the use of the Efron method for analysing ties in the data
Prentice, Williams and Peterson Total Time	*stset End, failure(Failure==1) id(ID) origin(time Clinic) enter(time Clinic) scale(365.25) time0(Start) exit(time.)*	*time0(Start) *specifies that 'Start' is the beginning of the time interval spanned by each record	*stcox varlist, efron robust strata(Risk_set)*	*robust *specifies that robust variance estimation is used to account for within-subject correlation of failure times
		*exit(time.) *ensures that an individual’s failures after the first are also recognised		*strata(Risk_set) *ensures that a stratified cox model is fitted with a different underlying risk for each ordered failure event

## Application of techniques to an analysis of the relationship between housing tenure and risk of hospital admission in the HCS-HES dataset

### Methods

Data were described using means and standard deviations (SD), medians and inter-quartile ranges (IQR) and frequency and percentage distributions. The association between housing tenure and risk of hospital admission or death was analysed using the following techniques: time to first event Cox regression; the Andersen and Gill (AG) model; and the Prentice, Williams and Peterson Total Time (PWP-TT) model. Analyses were conducted without and with adjustment for age, height, weight adjusted for height, smoking history, alcohol, and walking speed. We analysed different types of hospital admission: any admission; emergency admission; elective admission and long admission (greater than 7 days). All analyses were conducted for men and women separately using the syntax commands presented in Table 3 and using release 13.0 of the Stata statistical software package [22].

### Characteristics of HCS participants

Characteristics of the HCS study participants are shown in Table 4. The average age of the men and women at HCS baseline clinic was 66 years. Men were more likely to be ever smokers and to report a high weekly alcohol intake than women, but men and women had similar BMI, self-reported walking speed and number of systems medicated. 19 % of men and 22 % of women did not owner-occupy their home. In total, 1185 (75 %) men and 976 (68.8 %) women had experienced at least one hospital admission subsequent to their HCS baseline clinic but prior to 31st March 2010. Of the 189 men and 86 women who died during the follow-up period, only 12 men and 9 women died without having also experienced a hospital admission. In total, the HCS participants experienced 8687 hospital admissions of which 6462 were elective and 2201 were emergency admissions.

**Table 4:** Characteristics of HCS participants

n(%)	Men (*n* = 1579)	Women (*n* = 1418)
Age (yrs)^a^	65.7 (2.9)	66.6 (2.7)
Height (cm)^a^	174.2 (6.5)	160.8 (5.9)
Weight (kg)^a^	82.4 (12.7)	71.4 (13.4)
BMI (kg/m^2^)^a^	27.2 (3.8)	27.6 (4.9)
Ever smoked regularly	1059 (67.1 %)	553 (39.0 %)
High alcohol intake (≥22 M; ≥15 F units per week)	340 (21.5 %)	68 (4.8 %)
Home ownership (Not owned or mortgaged)	299 (18.9 %)	313 (22.1 %)
Walking speed (self-reported)^a^:		
Very slow	76 (4.8 %)	97 (6.8 %)
Stroll	375 (23.8 %)	285 (20.1 %)
Normal	625 (39.6 %)	638 (45.0 %)
Brisk	432 (27.4 %)	319 (22.5 %)
Fast	69 (4.4 %)	79 (5.6 %)
Number of systems medicated^b^	1.0 (0.0, 2.0)	1.0 (1.0, 2.0)
Deaths	189 (12.0 %)	86 (6.1 %)
Ever had an admission	1185 (75.0 %)	976 (68.8 %)
Ever had an admission/died	1197 (75.8 %)	985 (69.5 %)

*SD* standard deviation

^a^Mean(SD) ^b^Median (Lower quartile, Upper quartile)

### Associations between housing tenure and risk of hospital admission

Table 5 shows the association between housing tenure and the risk of different types of hospital admission as analysed by the time to first event Cox, AG, and PWP-TT models; the same broad conclusions were drawn from all analysis techniques. Among women, not owner-occupying one’s home was associated with increased risk of all types of hospital admission or death, irrespective of the survival analysis technique implemented: all associations except those between housing tenure and long or elective hospital admission or death remained significant after adjustment for potential confounders. Among men, not owner-occupying one’s home was associated with increased risk of emergency or long hospital admission or death according to all survival analysis techniques, although only the association between housing tenure and emergency admission was robust to adjustment for potential confounders.

**Table 5:** Associations between housing tenure and risk of hospital admission for different types of survival models

Failure event	Model Type	Men				Women			
		Unadjusted		Adjusted^a^		Unadjusted		Adjusted^a^	
		Hazard ratio	*P*-value	Hazard ratio	*P*-value	Hazard ratio	*P*-value	Hazard ratio	*P*-value
		(95 % CI)		(95 % CI)		(95 % CI)		(95 % CI)	
Any admission	TFEC	1.28 (1.11,1.47)	0.001	1.16 (1.00,1.34)	0.051	1.36 (1.18,1.57)	<0.001	1.19 (1.02,1.39)	0.025
	AG	1.20 (1.02,1.43)	0.030	1.07 (0.91,1.27)	0.414	1.36 (1.14,1.61)	<0.001	1.24 (1.04,1.47)	0.014
	PWP	1.00 (0.92,1.08)	0.921	0.96 (0.88,1.04)	0.306	1.16 (1.07,1.27)	0.001	1.12 (1.03,1.22)	0.010
Emergency admission	TFEC	1.63 (1.36,1.95)	<0.001	1.41 (1.17,1.70)	<0.001	1.67 (1.36,2.04)	<0.001	1.43 (1.16,1.77)	0.001
	AG	1.70 (1.37,2.12)	<0.001	1.45 (1.16,1.81)	0.001	1.66 (1.31,2.11)	<0.001	1.39 (1.09,1.77)	0.008
	PWP	1.23 (1.07,1.41)	0.003	1.15 (1.00,1.32)	0.043	1.34 (1.15,1.56)	<0.001	1.23 (1.06,1.42)	0.007
Elective admission	TFEC	1.16 (1.00,1.35)	0.047	1.05 (0.90,1.23)	0.523	1.30 (1.12,1.52)	0.001	1.15 (0.98,1.34)	0.090
	AG	1.05 (0.86,1.27)	0.639	0.94 (0.77,1.14)	0.515	1.28 (1.07,1.53)	0.008	1.19 (1.00,1.43)	0.052
	PWP	0.92 (0.84,1.00)	0.061	0.87 (0.79,0.95)	0.003	1.18 (1.07,1.30)	0.001	1.13 (1.03,1.24)	0.011
Long admission (>7 days)	TFEC	1.46 (1.18,1.80)	<0.001	1.19 (0.96,1.48)	0.114	1.47 (1.16,1.86)	0.001	1.18 (0.92,1.51)	0.187
	AG	1.49 (1.19,1.87)	0.001	1.18 (0.93,1.49)	0.166	1.40 (1.09,1.81)	0.010	1.12 (0.87,1.44)	0.388
	PWP	1.25 (1.05,1.48)	0.011	1.07 (0.89,1.28)	0.489	1.31 (1.07,1.60)	0.008	1.14 (0.93,1.40)	0.216

Estimates of associations are hazard ratios representing the increase in risk of the failure event among individuals who do not own/mortgage their home compared to individuals who do

*TFEC* time to first event Cox model, *AG* Andersen and Gill model, *PWP* Prentice, Williams and Peterson total time model

^a^Models were adjusted for age, height, weight adjusted for height, smoking status, weekly alcohol intake and self-reported walking speed

In spite of obtaining broadly similar conclusions about the pattern of association between housing tenure and risk of hospital admission or death from all survival analysis techniques, the hazard ratios estimated by the PWP-TT model were smaller than those from the time to first event Cox model and the AG model. For example, the time to first event Cox model estimated hazard ratios of 1.67 (95 %CI: 1.36, 2.04) and 1.63 (95 %CI: 1.36, 1.95) for the association between not owner-occupying one’s home and the risk of emergency admission or death among women and men respectively; the corresponding hazard ratios as estimated by the PWP-TT model were only 1.34 (95 %CI: 1.15, 1.56) for women and 1.23 (95 %CI 1.07, 1.41) for men.

## Discussion

Linkage between the HCS database and HES data has created a rich but complex multiple-failure survival dataset for the investigation of risk factors for hospital admission among older people; other UK cohorts are well placed to link with HES. This paper serves as a ‘toolkit’ to assist researchers in the appropriate analyses of multiple-failure survival datasets by: reviewing suitable analysis techniques; outlining their implementation using Stata; and contrasting their application in an indicative analysis of housing tenure as a socioeconomic risk factor for hospital admission. We recommend the Prentice, Williams and Peterson Total Time (PWP-TT) model for the analysis of multiple-failure survival datasets which detail hospital admissions among older people.

Our observation that the PWP-TT model gives smaller estimated hazard ratios than the time to first event Cox or Andersen and Gill models is consistent with previous research which investigated risk factors for hospital readmission in Brazil [23]. The PWP-TT model is likely to yield more conservative hazard ratios because it accounts for the underlying increase in risk of admission with the number of accumulated previous admissions. Failure to account for an increase in this underlying risk of admission may result in exaggerated estimates of the impact of a risk factor on hospital admission.

This paper has some limitations. First, we regarded hospital admission and death as equivalent failure events. This approach was necessitated because death cannot simply be regarded as a non-informative censoring event such as emigration or the end of follow-up [24]. Moreover, although competing risk regression [25], as an extension of time to first event Cox modelling, could account for deaths as a competing event (an event which occurs instead of the failure event of interest) and would be important to consider in a time to first event analysis of nursing home admission among elderly people where the risk of mortality is high, this approach is not extendable to multiple-failure survival datasets using routine statistical software. Second, our review of suitable techniques for multiple-failure survival datasets was focused on those that may be implemented using routine statistical software. Alternative techniques not discussed in this paper include; multi-state models, which investigate the relationship between individual risk factors and the transition probabilities between states representing different failure events [26]; frailty models, which are similar to a Cox’s proportional hazards model but include random effects to account for the within-subject correlation of failure times [17]; and the Wei, Lin and Weissfeld Model (WLW) model which has similarities to the AG and PWP-TT model but is poorly suited to the analysis of ordered failure events because the individual is regarded at risk of all repeated events from the outset [27].

This paper also has many strengths. First, we provide researchers with a comprehensive ‘toolkit’ for the analysis of multiple-failure survival datasets arising from linkage between cohort study datasets and routinely collected data on hospital admissions. We describe all stages of statistical analysis from the appropriate organisation of the dataset, to an understanding of the key properties of available analysis techniques and their implementation in Stata, through to a comparison of results from an indicative analysis of risk factors for hospital admission. This paper is a valuable resource which will enable researchers to apply complex multiple-failure survival analysis techniques in their own research. Second, our indicative analysis of the association between housing tenure and hospital admission used data from a well characterised cohort study of community-dwelling older men and women; data were collected by trained research doctors and nurses according to strict measurement protocols [8]. We therefore have confidence in the broad conclusion that not owner-occupying one’s home, an indicator of socioeconomic disadvantage, is associated with increased risk of hospital admission and this is consistent with the wide evidence base for a social gradient in health [28, 29].

## Conclusions

We recommend the Prentice, Williams and Peterson Total Time model for the analysis of multiple-failure survival datasets which detail hospital admissions among older people. This article serves as a toolkit to assist researchers in the appropriate analysis of multiple-failure survival datasets arising from data linkage between a cohort study and routinely collected data on hospital admissions.

### Availability of supporting data

We welcome opportunities for collaboration. Enquiries should be directed to Professor Cyrus Cooper, Director of the MRC Lifecourse Epidemiology Unit and Hertfordshire Cohort Study Principal Investigator, University of Southampton (cc@mrc.soton.ac.uk).
